# Clinical trials and pregnancy

**DOI:** 10.1038/s43856-022-00198-1

**Published:** 2022-10-25

**Authors:** Catriona Waitt, Denise Astill, Eleonor Zavala, Ruth A. Karron, Ruth R. Faden, Pamela Stratton, Sarah M. Temkin, Janine A. Clayton

**Affiliations:** 1 University of Liverpool, Liverpool, UK; 2 Foetal Anti-Convulsant Syndrome New Zealand, PO Box 82-175, Highland Park, Auckland, 2143 New Zealand; 3 Consumer Advocacy Alliance, PO Box 32 445, Devonport, Auckland, 0744 New Zealand; 4 Department of International Health, Bloomberg School of Public Health, Johns Hopkins University, 615 N. Wolfe St, Baltimore, MD 21205 USA; 5 Berman Institute for Bioethics, Johns Hopkins University, 1809 Ashland Avenue, Baltimore, MD 21205 USA; 6 Office of Research on Women’s Health, National Institutes of Health, Building 10, Room 7-4647, 10 Center Dr., Bethesda, Maryland 20892 USA

**Keywords:** Reproductive disorders, Toxicology, Reproductive biology

## Abstract

Traditionally, there has been a reluctance to involve pregnant people in clinical trials due to complex ethical issues surrounding the risk to unborn babies. However it is crucial that new interventions are safe and effective for all patients and ensuring this can be difficult to achieve in the absence of clinical trials.

In this Viewpoint, those with an interest in treatments required by pregnant people discuss the importance of undertaking clinical research on pregnant people and the considerations when undergoing such research.

## Catriona Waitt

**Figure:**
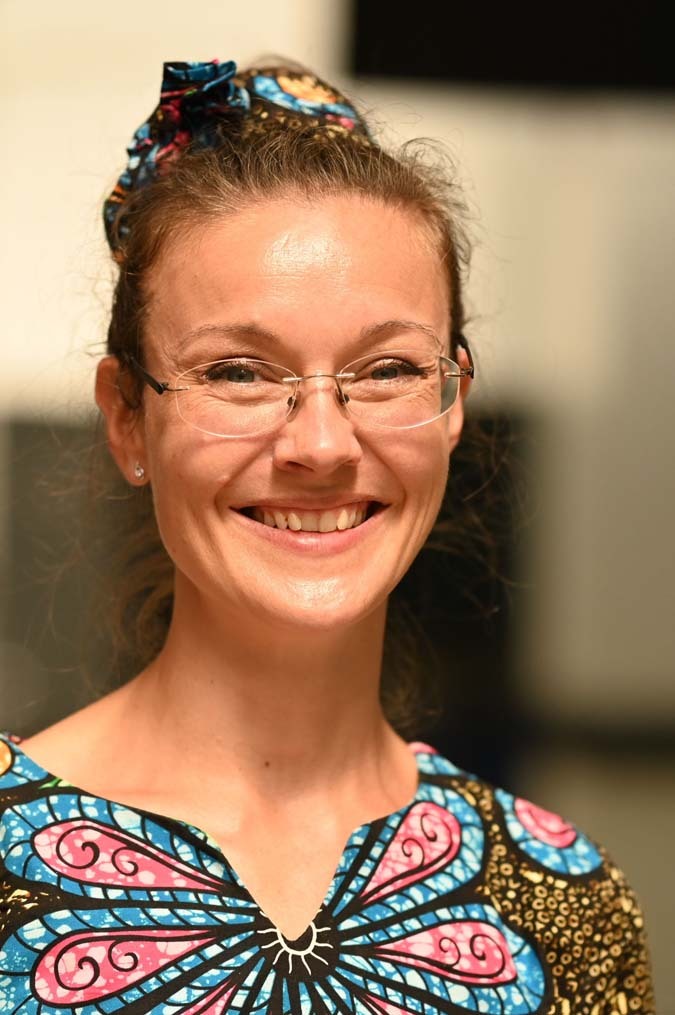
© Tabu Studios (@TabuCapital)

I am a Professor in Clinical Pharmacology and Global Health from the University of Liverpool, resident in Uganda since 2014 as a Wellcome Clinical Research Career Development fellow. My fellowship aims to provide evidence for breastfeeding women with a range of conditions to make informed decisions about medication use in breastfeeding.

Individuals with chronic medical conditions requiring drug treatment become pregnant and may wish to breastfeed, and those who are already pregnant or breastfeeding develop new medical conditions that require treatment with drugs. Worldwide, it is estimated that the majority of women take some form of medication during pregnancy. Pregnancy-induced changes in drug absorption, distribution, metabolism and elimination (pharmacokinetics) mean that in some cases the dose must be adjusted to ensure effect. The United States Food and Drug Administration has long stated that if a drug is anticipated to be widely used in those of childbearing age, that it should be studied in pregnancy and lactation around the time of licensing, but in reality this is seldom done^1^. However, such guidance is not mandated by law, and there remains little incentive for the pharmaceutical industry to sponsor such studies. Furthermore, many stakeholders including clinicians, researchers and regulatory bodies are reluctant to research in what they consider to be a high-risk area.

The concerns about performing clinical trials in pregnancy are justified as some drugs are known to be harmful to the unborn child, and the protection of this individual who cannot make an informed choice is paramount. However, there is a logical misperception that not performing such trials reduces the risk to mother and unborn child to zero. Worldwide, at least a third of pregnancies are unplanned, and in many regions of the world, late presentation to antenatal care is common. Furthermore, individuals and their clinicians may face the difficult choice of whether to use a potentially beneficial drug off label, without the pregnancy-specific evidence required. If a drug is prescribed to those with childbearing potential in a population, pregnancy exposures will occur, but outside a trial context these will not be carefully scrutinised and there may be a delay in recognising any adverse effects. This concept is referred to as risk shifting. Indeed, it could be argued that in the case of thalidomide, in which birth defects occuured in the children of women treated with the drug, a clinical trial would have identified the severe fetotoxicity far earlier and overall significant harms could have been averted^2^.

Everybody deserves the evidence upon which to base informed decisions about their health, and this requires such evidence to be generated. Not having this evidence can result in unfairness in the distribution of benefits and burdens (injustice) and can curtail the autonomy in making informed choice. This may make it impossible to provide the best treatment, undermining the principle of beneficence, and risking increasing harm. This goes against the principle of non-maleficence which underpins much clinical practice. In my opinion, inclusion of pregnant women in clinical trials, in situations where those of childbearing potential are likely to receive a medication, is therefore ethically justified.

The decision to include pregnant populations in clinical trials must never be made lightly. A step-wise approach should be used. Preclinical, animal model and healthy volunteer data are required before moving to any clinical trial. Risks and benefits must be scrutinised in each case, with initial clinical trials focussing on those who require specific medical treatment for their own health. Examples of best practice can be found in prevention of mother to child transmission of HIV. The only way to prevent the lifelong morbidity and risk of mortality among infants infected by their mothers is to treat during pregnancy, and therefore there was a strong driver to undertake studies in pregnancy from the earliest days of antiretroviral therapy. In addition to clinical trials, there have been a greater number and higher quality of pharmacokinetic studies of antiretrovirals in pregnancy compared to other classes of drug. Furthermore, there has been a strong voice from affected communities and strong partnerships and stakeholder relationships prioritising the generation of evidence in this field. In 2020, the international and interdisciplinary Pregnancy and HIV/AIDS: Seeking Equitable Study (PHASES) Working Group produced detailed guidance^3^ advancing 12 specific, measurable, actionable recommendations, based on extensive qualitative research, stakeholder engagement, expert consultation and a series of workshops. Other medical disciplines can draw from this pragmatic, robust, comprehensive guidance.

Although research inclusion of pregnant populations can be complex, the weighing of risks and benefits in daily, largely off-label treatment and clinical care is fraught with even greater complexity. Principles of bioethics such as autonomy dictate that those who are pregnant should be able to make their own decisions about participation in clinical research. Justice and beneficence demand that they have equitable access to new technologies and therapies that emerge from that research. Such research is ethically justified, and there exists clear guidance to support best practice in study design and conduct.

## Denise Astill

**Figure:**
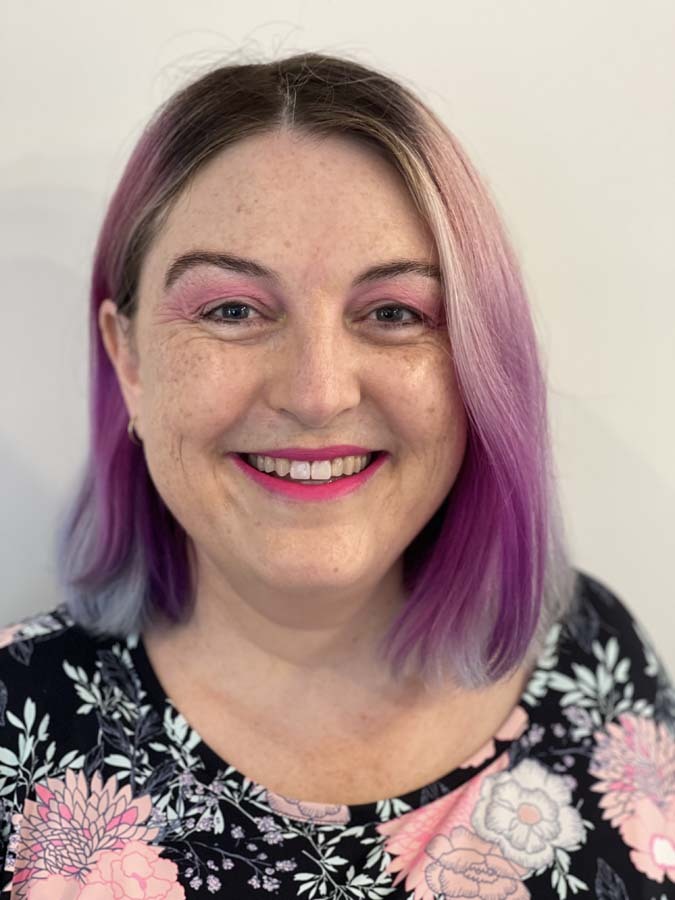
© the Author

As a woman with epilepsy I learnt early on that my seizures could be unpredictable. So when I decided to try for a baby I consulted with both a neurologist and a maternal medicine specialist to ask if my medication, sodium valproate, would be safe to an unborn baby. I was advised that if I took my medication there would be a 1–2% chance of spina bifida or neural tube defects, but to take folic acid and the baby would be fine. Alternatively I could come off the medication, but I would have seizures and the baby would be oxygen deprived, and therefore brain damaged. To me the choice was easy, I took my medication and folic acid as I was assured everything would be fine. Unfortunately that was not the case. Now I speak as a woman who has epilepsy, did not have informed consent or choice, and is raising two adult children who have Fetal Valproate Spectrum Disorder. Life is hard, and the guilt I carry is always there, sometimes dormant, sometimes outwardly present. If clinical trials for pregnant people were an option when I was pregnant I do not know if I would have participated. This is because as a consumer I simply did not have the information to fully understand, and give informed consent. Now though, I participate in, support, and help co-design research, from an expert-by-experience point of view, so future generations have the information that my adult children and I did not have.

When you are a person of childbearing potential, who is on an anti-seizure medication, sitting in an appointment with your specialist, more often than not, there is an unspoken hierarchal order in which you are 100% reliant on that healthcare professional. You are reliant on them giving you all the information you require to be fully informed and to be able to provide informed consent and informed choice. But what if they do not offer this to you, or do not know all the relevant information? Unfortunately, some childbearing people are told to Google whether their medication is safe in pregnancy!

We collectively need to challenge the status quo of this disparity, particularly if the childbearing person has a chronic health condition where research is limited. In reality, the childbearing person may not know to ask or query what their medication could do to an unborn baby, and will probably not know about clinical trials, let alone clinical trials in pregnancy. The fact they are sitting with the specialist possibly asking questions about their medication in pregnancy indicates they are concerned about their baby, or possible pregnancy in the future. Additionally, they are probably weighing up what might happen should they stop taking their medication, for example the possibility of a seizure, or recurrence of their mental health condition.

Consumers must become co-designers for any type of research or clinical trials. They are experts-by-experience and bring a wealth of knowledge that is not learnt in books. Often they are discovering topics that need to be thought about, researched, or developed, purely because it is something they are living and breathing. It is necessary to make sure, however, that this co-design is done in an accessible manner.

Education about clinical trials in pregnancy needs to be a common conversation, in an easy to understand manner. The childbearing person may want to be of assistance, but not comfortable with being in a clinical trial, so additional options need to be provided, such as participating in a pregnancy register/registry, so there is a need to know what other options are out there to provide this to the consumer.

Collectively, we can change the future of how clinical trials, research, and education is being provided through co-design, accessibility, and informed consent and choice. Through this collaboration the possibilities are limitless.

## Eleonor Zavala

**Figure:**
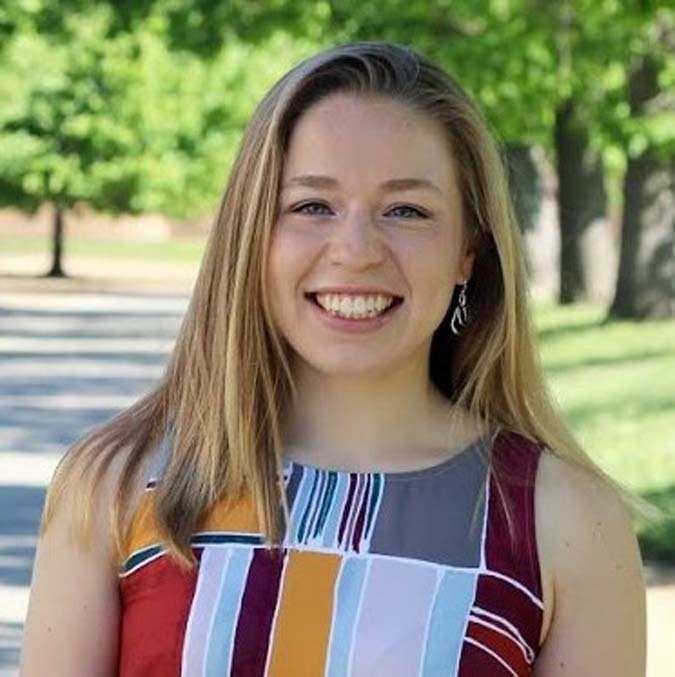
© the Author

## Ruth Karron

**Figure:**
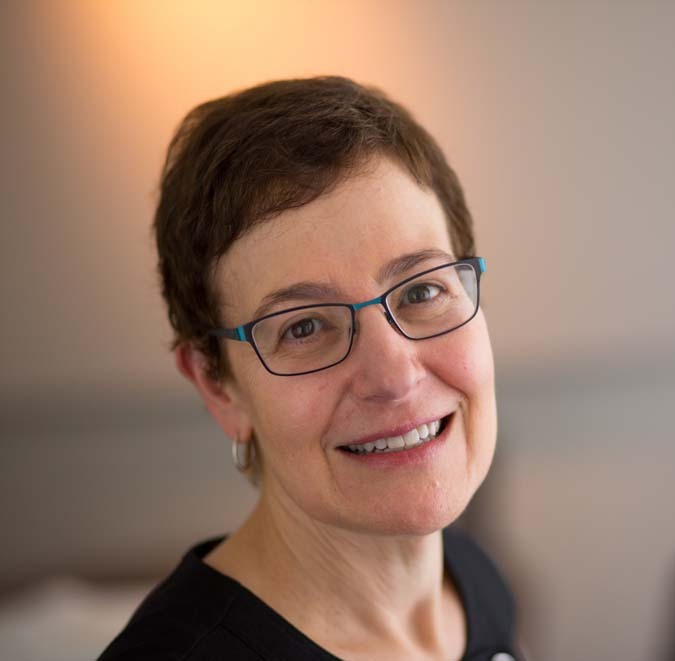
© the Author

## Ruth Faden

**Figure:**
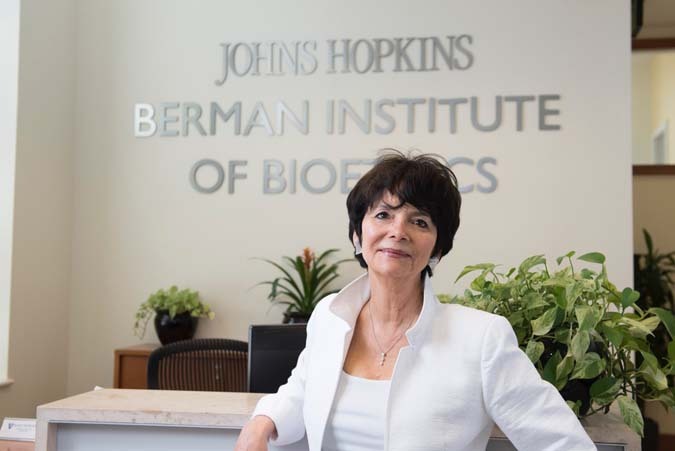
© the Author

We are all interested in maternal immunization, mostly recently in COVID-19 vaccines for pregnant and lactating people. Our backgrounds are in bioethics and health policy (RF), pediatric infectious disease and vaccinology (RK) and preventative approaches to maternal and child health (EZ).

Pregnant people have been continuously left behind in drug and vaccine development. Historically, pregnant people have been excluded from clinical trials because of concerns about legal liability and confusion about what is permissible from a regulatory and ethical perspective. In recent years, however, major shifts have occurred in thinking about the ethics of research in pregnancy. National and international groups have come to a consensus view that pregnant people and their offspring need to be protected through research, not from research^3,4^. As a matter of justice, pregnant people deserve both an evidence base for the prevention and treatment of illnesses equal to other adults, and fair access to participation in research that offers a prospect of direct benefit without undue risks to either the pregnant person or the fetus.

Despite these advances, pregnant people have continued to be excluded in many research contexts, including the development of vaccines and treatments for COVID-19^5^, in which pregnant people were excluded from phase 3 COVID-19 vaccine trials. Also, developmental and reproductive toxicology (DART) studies, a frequent prerequisite for the inclusion of pregnant people in clinical trials, either occurred late in development or were never conducted for certain products. The consequent lack of vaccine safety data was cited by a majority of countries as a reason for restricting or limiting access to COVID-19 vaccines for pregnant people^6^. This was especially apparent in low-and-middle-income countries (LMICs), where lack of data and limited vaccine supply led countries to adopt restrictive policies on the use of COVID-19 vaccines in pregnancy, or to have no policy at all^6^. However, pregnant women infected with SARS-CoV-2 have a greater risk of hospitalization, admission to the intensive care unit (ICU), invasive ventilation, need for oxygenation, and death compared to non-pregnant women infected with SARS-CoV-2^7^. Additionally, pregnant women who are infected with SARS-CoV-2 are at greater risk for pregnancy-specific adverse events than pregnant women who are not infected^8^. At the same time, real-world studies have demonstrated that the administration of COVID-19 vaccines in pregnancy does not increase the risk of these adverse events;^9^ moreover, vaccination protects both mother and infant against severe disease and hospitalization, providing dual benefit^10,11^.

While most countries have now adopted recommendations for the use of COVID-19 vaccines in pregnancy (https://www.comitglobal.org/), some countries continue to limit access and certain vaccines still do not have adequate safety data in pregnancy. While it is impossible to calculate how many pregnant people and their newborns were harmed by the initial and persistent delays in access to COVID-19 vaccines during pregnancy, there is no question that significant suffering could have been prevented.

Although the current pandemic is far from over, we are now facing a new infectious disease crisis. On July 23rd, 2022, the Director-General of WHO declared the recent outbreak of monkeypox a public health emergency^12^. Pregnant people may be at increased risk of severe disease. Moreover, transmission from a pregnant person to a fetus or newborn is possible and newborns are at risk of severe disease. No monkeypox vaccines are currently approved for use in pregnancy, and should vaccines need to be deployed in groups that include pregnant people, the absence of data specific to pregnancy may once again lead to policy decisions that restrict pregnant people’s access to a needed vaccine^13^.

There is now considerable momentum, spurred by the failures of the COVID-19 pandemic, to consider the needs of pregnant people and their offspring in pandemic preparedness as well as at the outset of an epidemic or pandemic. This momentum extends beyond the pandemic context, recognizing the importance of providing pregnant people, clinicians, and policymakers with an adequate evidence base for decision-making across the spectrum of threats to health. Key to securing this evidence base is the inclusion of pregnant people in vaccine and clinical treatment trials. Frameworks to guide the responsible development and deployment of vaccines and antiviral medications for pregnant people are readily available^3,4^. These frameworks provide substantive guidance on how to design trials for the ethical inclusion of pregnant people as well as procedural recommendations about the experts and advocates that need to be engaged as drug and vaccine development plans are being constructed. It is heartening to note that pregnant women are currently being included in at least one monkeypox treatment trial (NCT05534984). To protect pregnant people and their offspring against current and future infectious disease and other health threats, concerted global effort and political will are needed to make these frameworks operational.

## Pamela Stratton

**Figure:**
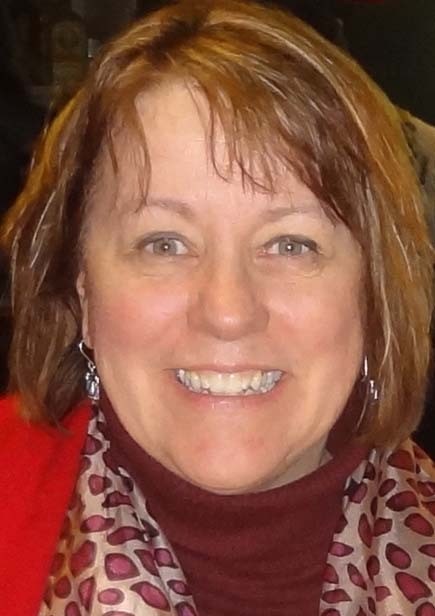
© the Author

## Sarah Temkin

**Figure:**
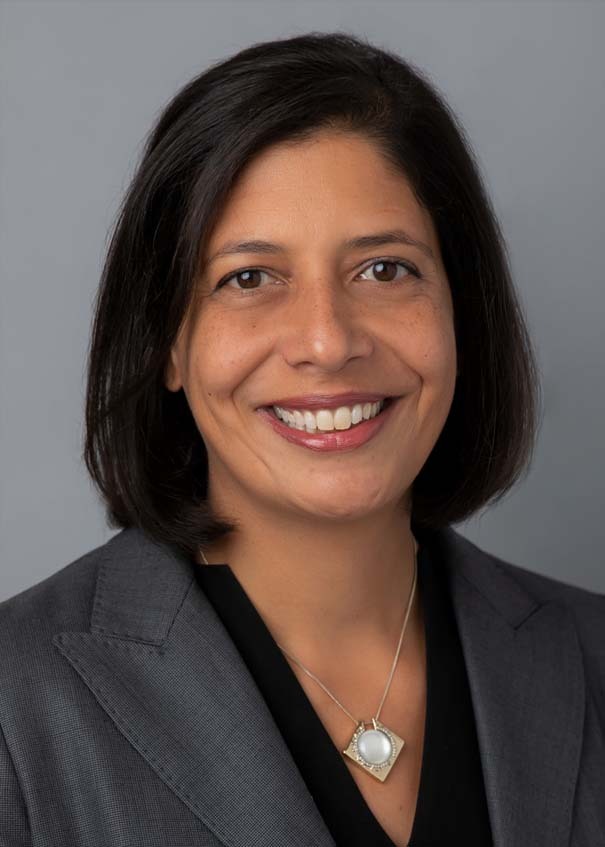
© the Author

## Janine Clayton

**Figure:**
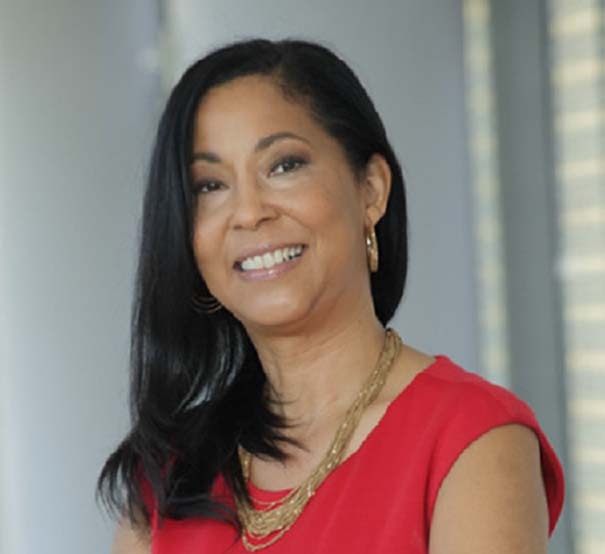
© the Author

As employees of the Office of Research on Women’s Health we have identified the research needed to improve maternal health in the US, including responding to a Congressional-mandated examination of NIH funding in women’s health. The COVID pandemic gave us an additional perspective on the issue of inclusion of pregnant people in research

Throughout the current COVID-19 pandemic, the National Institutes of Health (NIH) has coordinated, catalyzed and transformed scientific advances, translating clinical trials into therapies through rapid, worldwide, strategic multi-pronged collaborations. The NIH Office on Research for Women’s Health, which coordinates research on women’s health across the NIH’s Institutes, Centers and Offices, has been particularly attuned to the burden among Black and Indigenous women of social inequity in addition to pandemic consequences, that has been associated with a nearly four-fold risk of pregnancy-related death compared to non-Hispanic white people^14^. Pregnancy was a routine exclusion criteria for COVID-19 vaccine clinical trials despite a greater risk of morbidity and mortality associated with COVID-19 infection acquired during pregnancy. Even after real world evidence enabled COVID-19 vaccination to be recommended during pregnancy, vaccine hesitancy persisted, in part due to limited evidence of safety and efficacy.

The profound physiological changes that occur during pregnancy are a stress test for lifelong health, including infection, cardiovascular and metabolic diseases, as well as mental health conditions. Yet we lack fundamental information about the safety, dosage, and efficacy of nearly all therapeutics and vaccines administered during pregnancy. This gap arises from a historic categorization of those capable of pregnancy as members of a vulnerable population, in need of protection from clinical trials, and a lack of consideration of sex as a biological variable in the development of therapeutic agents. However, the scientific complexity and unique physiology of pregnancy coupled with an ethical mandate for evidence-based treatment supports their intentional inclusion in clinical trials, perhaps with guidance from the recently released implementation plans from The Task Force on Research Specific to Pregnant Women and Lactating Women^15^. Importantly, a life course perspective considers the interaction of innate, biological and external, and social factors that influence pregnancy outcomes and shape women’s health outcomes across their lifespan.

The rising rates of severe maternal morbidity and mortality in the United States that increased further during COVID underscore the need for intentional clinical research during pregnancy^16^. Clinical research is needed to improve care during pregnancy and after delivery that will effectively manage pregnancy-related and underlying comorbidities, reduce known maternal health risk factors, improve identification and management of severe morbidity, and expand comprehensive, interdisciplinary research to reduce preventable maternal deaths. Implementation research is critically needed to assess how to best apply evidence-based, high-quality care, such as those described in safety bundles - collections of best practices that offer a framework to incorporate established guidelines into health care practice using a standard approach to pregnancy and postpartum care - to all pregnant persons. This could fulfill the goal of holistic, person-centered care, including to populations with overlapping and intersecting identities. Researchers must integrate input and guidance from communities and conduct studies that emphasize prevention and wellness to bring science to those most affected by maternal morbidity and mortality. Research should deliberately focus on assessing how overall health influences pregnancy and how complications and severe morbidity impact health after pregnancy and beyond the childbearing years. We can and should protect those individuals capable of pregnancy with, rather than from, clinical research.
